# Macrophages as drivers of an opportunistic infection

**DOI:** 10.15698/mic2017.10.595

**Published:** 2017-09-13

**Authors:** Annette C. Vergunst, Nazareth Lopez Carranza, Lili Zhang, Margarida C. Gomes, Yara Tasrini, Annemarie H. Meijer, David O’Callaghan

**Affiliations:** 1 VBMI, INSERM, Univ. Montpellier, Nîmes, France.; 2 Current address: Section of Molecular Biology, Division of Biological Sciences, University of California, San Diego, La Jolla, USA.; 3 Institute of Biology Leiden, Leiden University, Leiden, The Netherlands.

**Keywords:** Burkholderia cenocepacia, macrophages, intracellular bacteria, biofilms, opportunistic infections, cystic fibrosis, zebrafish, nosocomial infections

## Abstract

Opportunistic pathogens are a worldwide cause of mortality and morbidity, and infections with intrinsically antibiotic-resistant pathogens have a large clinical, social and economic impact. Bacteria belonging to the *Burkholderia cepacia* complex (Bcc), ubiquitous in natural and industrial environments, are notorious pathogens for individuals with cystic fibrosis (CF). In addition, *Burkholderia cenocepacia* is emerging as the culprit of non-CF related, sometimes fatal infections. Knowledge of the underlying infection mechanism of these pathogens is important for efficient treatment, however, to date not much is known about the lifestyle of Bcc bacteria during infection. In our recent study published in PLoS Pathogens, we provide experimental evidence that macrophages are critically important for proliferation of *B. cenocepacia*, and are major drivers of fatal pro-inflammatory infections in zebrafish larvae. This is in agreement with recent clinical information showing that *B. cenocepacia* is mainly localised in phagocytes in infected CF lungs. A predominant intramacrophage stage and a host-detrimental role for macrophages have major implications for treatment strategies of both CF and non-CF infections. Intracellular survival of bacteria traditionally classified as extracellular, including *Staphylococcus aureus* and *Pseudomonas aeruginosa*, is an emerging theme. Our finding that macrophages are essential for proliferation of *B. cenocepacia* in the host suggests a new paradigm for Bcc infections and urges the development of novel anti-infectious therapies to efficiently disarm these intrinsically antibiotic resistant facultative intracellular pathogens.

The *Burkholderia cepacia* complex (Bcc) currently counts 21 officially named similar, but genetically distinct bacterial species of Gram-negative bacteria. They can cause disease in plants and animals and are opportunistic pathogens for humans. These bacteria are ubiquitously present in nature and are highly tolerant to many different stress conditions, allowing them to survive in industrial and hospital environments, persisting on abiotic surfaces or in supposedly sterile medical solutions and even disinfectants. Their relatively large genomes support high adaptability and plasticity under stress conditions. The presence of a large number of efflux pumps and their membrane composition makes these bacteria highly resistant to many antibiotics used in clinical practice.

In clinical microbiology, bacterial biofilms are usually correlated with persistent and chronic infections, and cause major problems through colonized implants. Bcc bacteria produce strong biofilms on abiotic surfaces, and also in the presence of isolated cells including epithelial cells. This extracellular bacterial community lifestyle has therefore been suggested to play an important role in bacterial persistence and severity of CF lung infection, as shown unambiguously for *Pseudomonas aeruginosa*. Simultaneously, over the last two decades, unequivocal evidence has been obtained showing that these bacteria can survive within both professional and non-professional phagocytes *in vitro*. This may be due to their ability to survive in protozoa such as amoebae in the natural environment. Pioneering work from the Valvano lab has shown that Bcc bacteria survive and replicate in macrophages by avoiding cellular host defence mechanisms through interference with lysosomal maturation pathways, especially under CF conditions. These studies, together with those of many other research groups worldwide, suggested an important role for intracellular bacteria in pathogenesis contributing to their persistence, the pro-inflammatory character of the infection and their invasiveness. However, the contribution of intracellular bacteria to disease outcome *in vivo* has remained obscure. A clinical study 16 years ago from Sajjan, at that time in the Forstner lab, localized Bcc bacteria in macrophages in infected CF lungs. More recently, intriguing results from the Randell and Boucher labs identified Bcc bacteria in the late-stage CF lung predominantly as single-cell organisms within macrophages, and in mucus, but not in biofilm-like structures. Our recent study using zebrafish larvae supports this view, providing for the first time experimental evidence *in vivo* that macrophages are a critical site for Bcc replication and ensuing fatal pro-inflammatory responses (Figure 1).

**Figure 1:**
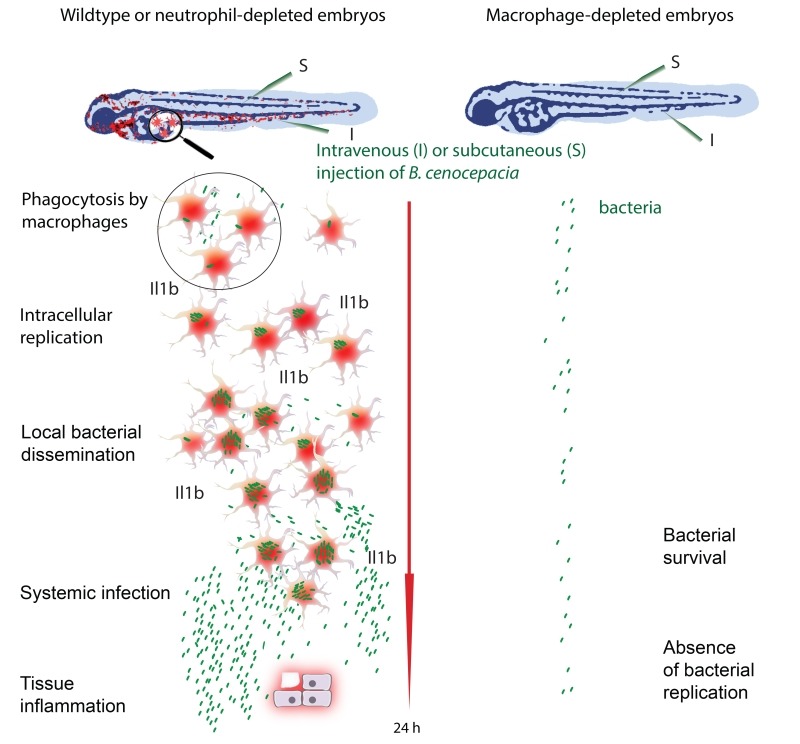
FIGURE 1: *B. cenocepacia* requires macrophages for efficient replication and the induction of acute pro-inflammatory disease. Zebrafish larvae are highly sensitive to infection with *B. cenocepacia* K56-2. Injection of a small inoculum (5 - 50 colony forming units) causes rapid pro-inflammatory fatal disease that requires the presence of macrophages, but not neutrophils. Macrophages are important for the observed increase in *il1b* expression. In the absence of macrophages, either using a Pu.1 morpholino knockdown approach or chemical ablation based on the Metronidazole / Nitroreductase system, *B. cenocepacia* K56-2 is unable to proliferate as in wildtype. This phenomenon was observed when the bacteria were injected intravenously and subcutaneously.

The zebrafish, *Danio rerio*, has become a popular biomedical research model, seen through the development of models of a wide variety of human diseases. The optically transparent zebrafish larvae are particularly amenable to study infectious disease mechanisms, making it possible to visualize the interaction between host phagocytes and pathogens in real time by non-invasive imaging. We used different methods to deplete zebrafish embryos of macrophages and found that Bcc strains that usually cause rapid death of the zebrafish host were considerably attenuated in virulence in the absence of these sentinels of the host immune system. This critical role for macrophages in bacterial replication and the onset of a robust, fatal pro-inflammatory response was seen when the bacteria were introduced intravenously as well as subcutaneously. Strikingly, in strong contrast, depletion of neutrophils had no measurable effect on disease outcome with both infection methods, despite the fact that subcutaneous infection is dominated by neutrophil-mediated phagocytosis. Thus, neutrophils do not contribute measurably to host defense or acute pro-inflammatory disease, emphasizing the detrimental effect of macrophages on the host in these acute fatal infections.

The zebrafish embryo model provides novel opportunities to investigate several outstanding questions concerning Bcc infections. We have found that while some Bcc strains, including several epidemic isolates, cause acute fatal infection, other strains multiply in macrophages but are mostly unable to escape from these immune cells, resulting in persistent infection with low pro-inflammatory responses. The reasons for these more silent infections are not known: do these bacteria avoid immune recognition, do they actively repress the host innate immune response, or can the host better control infections with these bacteria? We found that in the absence of macrophages, some of these less virulent, but persisting Bcc strains showed slightly increased bacterial burden, indicating that macrophages are not only critical for pro-inflammatory fatal responses with highly virulent strains but also contribute to control and degradation of less virulent bacteria.

In contrast to observations for pathogens such as *Salmonella* and *Mycobacterium*, we found that strains belonging to different Bcc species, causing either acute fatal or persistent infections, were unable to replicate in zebrafish larvae depleted of macrophages. This striking result suggests that Bcc bacteria are incapable of proliferating in an immune competent host, unless they encounter an intramacrophage environment. It is only after a first initial acute pro-inflammatory response in macrophage-proficient animals, and after several cycles of intracellular replication and local dissemination that the bacteria spread systemically and re-enter the blood circulation where they can then replicate extracellularly during a high pro-inflammatory state. Elucidating the bacterial and/or host factors that play a role in this phenomenon will be important for the design of both new therapeutic strategies and prognostic tests that will help with patient management.

During acute infections after *B. cenocepacia* challenge the host responded with robust pro-inflammatory cytokine expression in contrast to minor induction of gene expression during persistent infections caused by *B. stabilis*. We further found that the knockdown of *il1b* expression resulted in increased host mortality, while the Il1b antagonist Anakinra decreased host sensitivity to *B. cenocepacia* challenge inoculation. Thus, although *il1b* expression is needed for host defence, the balance of IL1 signaling is skewed towards host-detrimental inflammation. Our unpublished data further suggest that the inflammasome adaptor protein Apoptosis-Associated Speck-Like Protein Containing a CARD (ASC) also serves a beneficial role to the host during acute *B. cenocepacia* infections, since embryos infected with *B. cenocepacia* die faster in the absence of this inflammasome component. A role for inflammasome activation in host defence is in agreement with recent data from the Shao and Valvano labs that have shown that activation of the pyrin inflammasome by the type 6 effector TecA from *B. cenocepacia* has a protective role for mice. We are now further investigating the role of inflammasomes during both acute and persistent infections to elucidate which inflammasome components and signaling pathways contribute to the rapidly fatal infection in zebrafish larvae. Better knowledge of the factors contributing to host-protective and host-detrimental responses may help develop therapies to reduce the pro-inflammatory responses caused by these bacteria.

Bcc infections have mainly been described in the context of CF. Recent evidence is accumulating that these highly resistant opportunistic bacteria can also cause fatal non-CF related infections, both in- and outside the hospital, possibly caused by contaminated medical products or intravenous access. More information is needed, both from clinical studies and experimental animal models with a larger panel of isolates covering different Bcc species, to determine the contribution of macrophages during the different infection stages in CF and non-CF infections. Intracellular bacteria, hiding from the immune system and ectopically applied antibiotics, require different treatment strategies than bacteria residing in biofilms. Knowledge of the bacterial life style during infection *in vivo* is of utmost importance for development and efficient application of novel antimicrobial therapies. We believe the zebrafish model will be instrumental to help advance our knowledge of the role of phagocytes and the innate immune response in both CF and non-CF conditions.

